# *Oxydromus* Grube, 1855 reinstated over *Ophiodromus* Sars, 1862 (Polychaeta, Hesionidae)

**DOI:** 10.3897/zookeys.241.3820

**Published:** 2012-11-12

**Authors:** Tulio F. Villalobos-Guerrero, Leslie H. Harris

**Affiliations:** 1 Geomare A. C., Mazatlán, Sinaloa, México; 2 Natural History Museum of Los Angeles County, 900 Exposition Boulevard, Los Angeles, CA, 90007

**Keywords:** Nomenclature, taxonomy, hesionid, Phyllodocida, Annelida

## Abstract

The hesionid polychaete genera *Oxydromus* Grube, 1855 and *Ophiodromus* Sars, 1862 have been regarded as synonyms with the former considered as invalid since it was thought to be a junior homonym of *Oxydromus* Schlegel, 1854. However, Schlegel’s name is an incorrect subsequent spelling for *Ocydromus* Wagler, 1830 (Aves, Gruiformes, Rallidae) and is not an available name. Consequently, *Oxydromus* Grube, 1855 must be reinstated for this hesionid polychaete genus. A check-list of valid species of *Oxydromus* including 30 new combinations is provided.

## Introduction

Grube (1855) proposed *Oxydromus* within the polychaete family Phyllodocidae for *Oxydromus fasciatus* Grube, 1855, a new species capable of rapid movement from two Mediterranean Sea localities: Trieste (Italy) and Villa Franca (probably Villefranche-sur-Mer, France). Later, Sars (1862) established the genus *Ophiodromus* for a Norwegian species, *Ophiodromus vittatus* Sars, 1862. He also transferred *Oxydromus* to the family Hesionidae and distinguished it from *Ophiodromus* by the presence of articulated palps and biramous parapodia. Both features were present in *Ophiodromus fasciatus* but misinterpreted by Grube when he defined them as simple palps and uniramous parapodia (von Marenzeller 1874, Pleijel 2011 pers. comm.).

Pleijel (1998) examined syntypes of *Oxydromus fasciatus* (ZMB 3825), the type species of the genus, and specimens of *Nereis flexuosa* delle Chiaje, 1825 (currently *Ophiodromus flexuosus* fide Pleijel 1998) from near the type locality (type material of this species does not exist). He agreed with McIntosh (1908:116) that the former is a junior synonym of the latter although he didn’t go into detail. Nevertheless, regarding the defining generic characters, *Ophiodromus* and *Oxydromus* are synonymous. *Ophiodromus flexuosus* is possibly a senior synonym of the type species *Ophiodromus vittatus* (fide von Marenzeller 1874, McIntosh 1908, Pleijel 1998, Fauchald 2011). An examination of specimens from the type localities, Gulf of Naples and Norway respectively, is required to resolve their status.

Pleijel (1998) pointed out that *Oxydromus* has seniority over *Ophiodromus* but, as first stated by Hartman (1965), concluded that the former genus name was preoccupied in the class Aves, *Oxydromus* Schlegel, 1854, and for which reason *Ophiodromus* must be used. However, Viéitez et al. (2004) argued that *Oxydromus* is an available genus name and must be considered as valid. Then, following their suggestion, we proposed to reinstate *Oxydromus* over *Ophiodromus* to standardize the worldwide use of both generic names.

## Results

Viéitez et al. (2004:521) realized that *Oxydromus* Schlegel is an incorrect subsequent spelling of *Ocydromus* Wagler, 1830 (Aves, Gruiformes, Rallidae) (itself a junior homonym of the beetle genus *Ocydromus* Clairville, 1806 [Insecta, Carabidae], and replaced by *Gallirallus* Lafresnaye, 1841). Although Schlegel’s name was used in later publications (e. g. Reischek 1886, Röse 1890), as an incorrect subsequent spelling it remains unavailable according to Article 33.3 of the International Code of Zoological Nomenclature. This states “Any subsequent spelling of a name different from the correct original spelling, other than a mandatory change or an emendation, is an ‘incorrect subsequent spelling’; it is not an available name and, like an incorrect original spelling [Article 32.4], it does not enter in homonymy and cannot be used as a substitute name…”. Viéitez et al. erroneously stated that Sars proposed *Ophiodromus* to replace *Oxydromus* Grube due to his mistaken belief that Grube’s genus name is a junior homonym of *Oxydromus* Schlegel. Sars actually didn’t mention the homonymy; instead he discussed the morphological discrepancy between both genera.

Preservation of the genus-group name *Ophiodromus* as the senior synonym would require a reversal of precedence according to Article 23.9 (ICZN 1999). This states that in order to maintain the prevailing usage the following conditions must be met: 1) the senior synonym (in this case *Oxydromus* Grube, 1855) has not been used as a valid name after 1899 (Article 23.9.1.1), and 2) the junior synonym (namely *Ophiodromus* Sars, 1862) has been used for a particular taxon, as its presumed valid name, in at least 25 works, published by at least 10 authors in the immediately preceding 50 years and encompassing a span of not less than 10 years (Article 23.9.1.2). The first condition is not applicable which negates the need for a reversal of precedence. *Oxydromus* has also been listed as a valid name after 1900 in many publications (e.g. Gravier 1900, McIntosh 1908, Chamberlin 1919, Fauvel 1923, Augener 1927, Uschakov 1955, Hartmann-Schröder 1959, 1965, Hartman 1961, Day 1963, Averincev 1972).

In accordance with Article 23.9.3 (ICZN 1999), we consider that the *Oxydromus*/*Ophiodromus* situation does not threaten the stability of nomenclature or the universality of a widespread use, thus no referral to the Commission for a ruling should be required. We propose to reinstate *Oxydromus* over *Ophiodromus* based on the Principle of Priority (Article 23, ICZN 1999).

## Systematics

### Family HESIONIDAE Grube, 1850. Subfamily OPHIODROMINAE Pleijel, 1998. Tribe OPHIODROMINI Pleijel, 1998

#### Oxydromus

Grube, 1855 reinstated

Oxydromus Grube, 1855: 98.
Ophiodromus Sars, 1862: 87; Pleijel, 1998: 137–143, figs. 31–33 (synonymy).

##### Type species.

*Oxydromus fasciatus* Grube, 1855, by monotypy.

*Oxydromus adorsosetosus* (Hartmann-Schröder, 1985), comb. n. (basionym of *Ophiodromus adorsosetosus* Hartmann-Schröder, 1985)

Type locality: Port Lincoln, South Australia.

*Oxydromus adspersus* (Grube, 1874), comb. n. (basionym of *Ophiodromus adspersus* Grube, 1874)

Type locality: Dalmatia, Croatia.

*Oxydromus agilis* (Ehlers, 1864), comb. n. (basionym of *Ophiodromus agilis* Ehlers, 1864)

Type locality: Adriatic Sea.

*Oxydromus angolaensis* (Hartmann-Schröder, 1974), comb. n. (basionym of *Podarke angolaensis* Hartmann-Schröder, 1974)

Type locality: Lobito, Angola.

*Oxydromus angustifrons* (Grube, 1878), comb. n. (basionym of *Irma angustifrons* Grube, 1878)

Type locality: Philippines.

*Oxydromus berrisfordi* (Day, 1967), comb. n. (basionym of *Ophiodromus berrisfordi* Day, 1967)

Type locality: Walvis Bay, Namibia.

*Oxydromus brevipodius* (Uchida, 2004), comb. n. (basionym of *Ophiodromus brevipodius* Uchida, 2004)

Type locality: Wakayama, Japan.

*Oxydromus bunbuku* (Uchida, 2004), comb. n. (basionym of *Ophiodromus bunbuku* Uchida, 2004)

Type locality: Shikoku, Japan.

*Oxydromus constrictus* (Uchida, 2004), comb. n. (basionym of *Ophiodromus constrictus* Uchida, 2004)

Type locality: Wakayama, Japan.

*Oxydromus didymocerus* (Schmarda, 1861), comb. n. (basionym of *Cirrosyllis didymocerus* Schmarda, 1861)

Type locality: New South Wales, Australia.

*Oxydromus fasciatus* Grube, 1855 (possible junior synonym of *Oxydromus flexuosus* (delle Chiaje, 1825))

Type locality: Adriatic Sea (Trieste) and Western Mediterranean Sea (Villefranche-sur-Mer, France).

*Oxydromus fauveli* (Uchida, 2004), comb. n. (basionym of *Ophiodromus fauveli* Uchida, 2004)

Type locality: Wakayama, Japan.

*Oxydromus flexuosus* (delle Chiaje, 1825) (basionym of *Nereis flexuosa* delle Chiaje, 1825)

Type locality: Gulf of Naples.

*Oxydromus furcatus* (Hartmann-Schroder, 1962), comb. n. (basionym of *Podarke furcatus* Hartmann-Schroder, 1962)

Type locality: Peru.

*Oxydromus guanicus* (Hoagland, 1919), comb. n. (basionym of *Podarke guanica* Hoagland, 1919)

Type locality: Guanica, Puerto Rico.

*Oxydromus latifrons* (Grube, 1878), comb. n. (basionym of *Irma latifrons* Grube, 1878)

Type locality: Philippines.

*Oxydromus limicolus* (Willey, 1905), comb. n. (basionym of *Irma limicola* Willey, 1905)

Type locality: Sri Lanka.

*Oxydromus longifundus* (Uchida, 2004), comb. n. (basionym of *Ophiodromus longifundus* Uchida, 2004)

Type locality: Okinawa, Japan.

*Oxydromus longicirratus* (Knox and Cameron, 1971), comb. n. (basionym of *Nereimyra longicirratus* Knox and Cameron, 1971; not a senior homonym to *Oxydromus longocirratus* (Tenerelli, 1973) as Pleijel (1998) suggested, but this name is considered as nomen dubium)

Type locality: Melbourne, Australia.

*Oxydromus microantennatus* (Hutchings and Murray, 1984), comb. n. (basyonymy of *Podarke microantennata* Hutchings and Murray, 1984)

Type locality: New South Wales, Australia.

*Oxydromus minutus* (Hartmann-Schröder, 1959), comb. n. (basyonymy of *Podarke minuta* Hartmann-Schröder, 1959)

Type locality: San Juan, El Salvador.

*Oxydromus mutilatus* (Treadwell, 1901), comb. n. (basyonymy of *Castalia mutilata* Treadwell, 1901)

Type locality: Puerto Rico.

*Oxydromus notospinosus* (Rosito, 1983), comb. n. (basionym of *Ophiodromus notospinosus* Rosito, 1983)

Type locality: Philippines.

*Oxydromus obscurus* (Verrill, 1873), comb. n. (basyonymy of *Podarke obscura* Verrill, 1873)

Type locality: Massachusetts, United States.

*Oxydromus okudai* (Uchida, 2004), comb. n. (basionym of *Ophiodromus okudai* Uchida, 2004)

Type locality: Nagasaki, Japan.

*Oxydromus pallidus* Claparède, 1864

Type locality: Golfe du Lion, France.

*Oxydromus parapallidus* (Uchida, 2004), comb. n. (basionym of *Ophiodromus parapallidus* Uchida, 2004)

Type locality: Wakayama, Japan.

*Oxydromus pelagicus* (Rioja, 1923), comb. n. (basionym of *Ophiodromus pelagicus* Rioja, 1923)

Type locality: Pontavedra, Spain.

**Figure 1.**
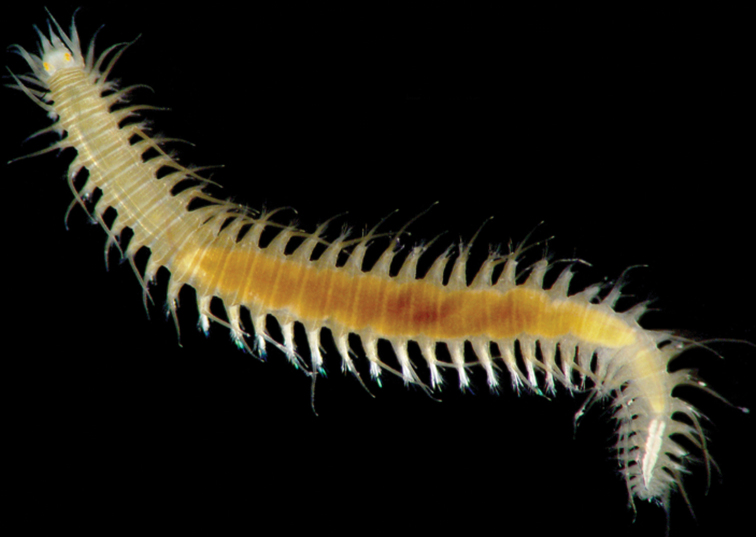
A representative living specimen of *Oxydromus* collected in California. Species: *Oxydromus pugettensis* (Johnson, 1901) (photo: Leslie Harris).

*Oxydromus pugettensis* (Johnson, 1901), comb. n. (basyonymy of *Podarke pugettensis* Johnson, 1901) (Figure 1)

Type locality: Washington, United States.

*Oxydromus spinapandens* (Storch and Niggemann, 1967), comb. n. (basyonymy of *Podarke pugettensis spinapandens* Storch and Niggemann, 1967)

Type locality: Red Sea.

*Oxydromus spinosus* (Ehlers, 1908), comb. n. (basyonymy of *Orthodromus spinosus* Ehlers, 1908)

Type locality: Angola.

*Oxydromus viridescens* (Ehlers, 1864), comb. n. (basyonymy of *Podarke viridescens* Ehlers, 1864)

Type locality: Adriatic Sea.

*Oxydromus vittatus* (Sars, 1862), comb. n. (basionym of *Ophiodromus vittatus* Sars, 1862)

Type locality: Norway.

## Remarks and discussion

Pleijel (1998) revised the phylogeny and classification of the family Hesionidae based on available type and non-type material. He provided descriptions and diagnoses for all supraspecific taxa and world-wide species lists, including 24 nominal taxa in *Ophiodromus*. He newly synonymized *Orseis* Ehlers, 1864 and *Schmardiella* Czerniavsky, 1882, and continued the prior synonymy of *Oxydromus* Grube, 1855, *Podarke* Ehlers, 1864, *Mania* de Quatrefages, 1866 and *Irma* Grube, 1878 with *Ophiodromus* Sars, 1862, whose type species is *Ophiodromus vittatus* Sars, 1862 (possibly *Ophiodromus flexuosus* fide Pleijel 1998). However, *Oxydromus* is herein reinstated due to the priority of *Oxydromus* over *Ophiodromus* Sars, with *Ophiodromus fasciatus* as the type species.

*Oxydromus* (from the Greek *oxys*= fast, quick; *dromus*= runner) (Figure 1) is currently represented by 32 species and is distinguished from other genera by the presence of six pairs of enlarged anterior cirri, prostomium bearing three antennae with the median one sited anteriorly, and biarticulated palps (see Salazar-Vallejo and Orensanz 2006). Uchida (2004) suggested that the species of this genus are among the most difficult groups to identify in the family and considered that the form of parapodia is a more useful character for identification of the species than prostomium and anterior part structures. A detailed revision of the genus *Oxydromus* is required to redefine species, especially those considered to be widely distributed.

## Supplementary Material

XML Treatment for Oxydromus

## References

[B1] Augener H (1927) Polychaeten von Neu-Pommern. Sitzungsberichte der Gesellschaft der naturforschende Freunde zur Berlin 1926: 119-152.

[B2] Averincev VG (1972) Benthic errant polychaetes from the Antarctic and subantarctic collected by the Soviet Antarctic Expeditions. Issledovaniye Fauny Moreyi 5: 88-293. [in Russian]

[B3] Chamberlin RV (1919) The Annelida Polychaeta. Memoirs of the Museum of Comparative Zoology at Harvard College 48: 1-514.

[B4] Clairville JP (1806) Entomologie Helvétique ou catalogue des insectes de la Suisse rangés d’après une nouvelle méthode [Helvetische Entomologie oder Verzeichniss der Schweizerischen Insekten nach einer neuen Methode geordnet]. Zweiter Theil. Zürich, Orell & Füssli, 251 pp., 32 pl.

[B5] Claparède É (1864) Glanures zootomiques parmi les Annélides de Port Vendres (Pyrénées Orientales). Mémoires de la Société de Physique et d’Histoire Naturelle de Genève 17: 463–600.

[B6] Czerniavsky V (1882) Materialia ad zoographiam Ponticam comparatam. Société Impériale des Naturalistes de Moscou 57: 146-198.

[B7] Day JH (1963) The polychaete fauna of South Africa. Bulletin of the British Museum (Natural History), Zoology 10: 381-445.

[B8] Day JH (1967) Polychaeta of Southern Africa. Part 1. Errantia. British Museum (Natural History), London, 458 pp.

[B9] Lafresnaye MF (1841) Nouvelles espèces d’oiseaux. Revue Zoologique (Société Cuvierienne) 4: 241-243.

[B10] de Quatrefages A (1866) Histoire Naturelle des Annelés Marins et d’Eau Douce: Annélides et Géphyriens. Volume 2. Première Partie. 1–336. Deuxième Partie. 337–794. Explication des planches p. 1–24. Planches 1–20. Collection des Suites a Buffon formant avec les Œuvres de cet auteur un Cours Complet d’Histoire Naturelle. Librairie Encyclopédique de Roret, Paris.

[B11] delle Chiaje S (1825) Memorie sulla storia e notomia degli animali senza vertebre del Regno di Napoli, Volume 2. Stamperia della Societa’ Tipografica, Napoli, 185–444. [plates issued in 1822]

[B12] Ehlers E (1864) Die Borstenwürmer, nach systematischen und anatomischen Untersuchungen dargestellt. Leipzig: Wilhelm Engelmann. doi: 10.5962/bhl.title.2081

[B13] Ehlers E (1908) Die bodensassigen Anneliden aus dern Sammlungen der deutschen Tiefsee-Expedition. Wissenschaftliche Ergebnisse der deutschen Tiefsee-Expedition auf dem Dampfer ‘Valdivia’ 1898–1899 16: 1–168.

[B14] Fauchald K (2011)*Ophiodromus vittatus* Sars 1861. In: Read G, Fauchald K (Eds) (2011) World Polychaeta database. http://www.marinespecies.org/polychaeta/aphia.php?p=taxdetails&id=340201

[B15] Fauvel P (1923) Polychètes errantes. Faune de France. 5. Librairie de la Faculté des Sciences. Paris, 488 pp.

[B16] Gravier C (1900) Contribution a l’étude des annélides polychètes de la Mer Rouge. Nouvelle Archives du Muséum d’Histoire Naturelle 2 (2): 137-282.

[B17] Grube AE (1855) Beschreibung neuer oder wenig bekannter Anneliden. Archiv für Naturgeschichte 21: 81-136.

[B18] Grube AE (1874) Ober seine im verflossenen August und September ausgefilhrte Reise nach der Kilste von Dalmatien. Jahres-Bericht der Schlesischen Gesellschaft fir vaterldndische Kultur 51: 52-56.

[B19] Grube AE (1878) Annulata Semperiana. Beiträge zur Kenntniss der Annelidenfauna der Philippinen nach den von Herrn Prof. Semper mitgebrachten Sammlungen. Mémoires Présentés a l‘Académie Impériale des Sciences de St. Petersburg (Séries 7) 25(8): 1–300.

[B20] Hartman O (1961) Polychaetous annelids from California. Allan Hancock Pacific Expeditions 25: 1-226.

[B21] Hartman O (1965) Supplement and index to the catalogue of the polychaetous annelids of the World, including additions and emendations since 1959. Occasional Papers of the Allan Hancock Foundation 23: 1-197.

[B22] Hartmann-Schröder G (1959) Zur Økologie der Polychaeten des Mangrove-Estero-Gebietes von El Salvador. Beiträge Zur Neotropischen Fauna 1 (2): 69-183. doi: 10.1080/01650525909380612

[B23] Hartmann-Schröder G (1962) Die Polychaeten des Eulitorals. In: Hartmann-Schröder G Hartmann G (Eds). Zur Kenntnis des Eulitorals der chilenischen Pazifikküste und der argentinischen Küste Südpatagoniens unter besonderer Berücksichtigung der Polychaeten und Ostracoden. Mitteilungen aus dem Hamburgischen zoologischen Museum und Institut 60: 57–270.

[B24] Hartmann-Schröder G (1965) Die Polychaeten des Sublitorals. In: Hartmann-Schröder G Hartmann G (Eds). Zur Kenntnis des Sublitorals der chilenischen Küste unter besonderer Berücksichtigung der Polychaeten und Ostracoden. Mitteilungen aus dem Hamburgischen zoologischen Museum und Institut 62: 59–305.

[B25] Hartmann-Schröder G (1974) Zur kenntnis des eulitorals der afrikanischen westküste zwischen Angola und Kap der Guten Hoffnung und der afrikanischen ostküste von Südafrika und Mocambique unter besonderer berücksichtigung der Polychaeten und Ostracoden. Teil II. Die Polychaeten des Untersuchungsgebietes 69: 95-228.

[B26] Hartmann-Schröder G (1985) Die Polychaeten der antiborealen Südküste Australiens (zwischen Port Lincoln im Westen und Port Augusta im Osten). Teil II. In: Hartmann-Schröder G Hartmann G (Eds). Zur Kenntnis des Sublitorals der chilenischen Küste unter besonderer Berücksichtigung der Polychaeten und Ostracoden. Mitteilungen aus dem Hamburgischen zoologischen Museum und Institut 82: 61–99.

[B27] Hoagland RA (1919) Polychaetous annelids from Porto Rico, the Florida Keys and Bermuda. Bulletin of the American Museum of Natural History 41: 517-591.

[B28] Hutchings P Murray A (1984) Taxonomy of the polychaetes from the Hawkesbury River and the southern estuaries of New South Wales, Australia. Records of the Australian Museum 36: 1-119.

[B29] International Commission on Zoological Nomenclature (1999) International code of zoological nomenclature (4 Edition). The International Trust for Zoological Nomenclature, London. 10.3897/zookeys.931.51583 PMC7205856 32405237

[B30] Johnson HP (1901) The Polychaeta of the Puget Sound region. Proceedings of the Boston Society of Natural History 29 (18): 381-437.

[B31] Knox GA Cameron DB (1971) Port Phillip Survey Pt. 2. 4. Polychaeta. Memoirs of the National Museum of Victoria 32: 21-41.

[B32] McIntosh WC (1908) A monograph of the British Annelids. Vol. 2, Pt. 1. Po1ychaeta. Nephthydidae to Syllidae. Ray Society, London.

[B33] Pleijel F (1998) Phylogeny and classification of Hesionidae (Polychaeta). Zoologica Scripta 27: 89-163. doi: 10.1111/j.1463-6409.1998.tb00433.x

[B34] Reischek A (1886) Ornithologische Beobachtungen aus Neu-Seeland. Mittheilungen des Ornithologisches Vereines in Wein 10 (10): 109-112.

[B35] Rioja E (1923) Nota acerca del género *Ophiodromus* Sars y descripción del *Ophiodromus pelagica* n. sp. Boletín de la Real Sociedad Española de Historia Natural 23: 217-224.

[B36] Rosito RM (1983) Polychaeta from shallow waters off Mactan, Cebu. The Philippine Scientist 20: 11-38.

[B37] Röse C (1890) Beitrage Zur vergleichenden Anatomie des Herzens der Wirbeltiere. Morphologisches Jahrbuch 16: 27-96.

[B38] Salazar-Vallejo SI Orensanz JM (2006)*Pleijelius longae* n. gen., n. sp., a remarkable deep water polychaete from the Northwestern Atlantic (Polychaeta: Hesionidae). Scientia Marina 70(S3): 157–166.

[B39] Sars M (1862) Uddrag af en af detaillerade Afbildningar ledsaget udførrlig Beskrivelse over føgende Norske Annelider. Forhandlinger i Videnskabs-Selskabet i Christiania 1862: 87-95.

[B40] Schlegel H (1854) Ook en woordje over den dodo (*Didus ineptus*) en zijne verwanten. Verslagen en Mededeelingen der Koninklijke Akademie van Wetenschappen 2: 232-256.

[B41] Schmarda LK (1861) Neue wirbellose Thiere beobachtet und gesammelt auf einer Reise un die Erdr 1853 bis 1857. Erster Band (zweite halfte) Turbellarian, Rotatorien un Anneliden. Wilhelm Engelmann, Leipzig, 164 pp.

[B42] Storch V Niggemann R (1967) Auf Echinodermen lebende Polychaeten. Kieler Meeresfomhungen 23: 156-164.

[B43] Tenerelli V (1973) Ricerche sulla fauna e sulla zoogeografia della Sicilia. 63. *Ophiodromus longocirratus* n. sp. (Polychaeta: Hesionidae) del Golfo di Catania. Bollettino delle sedute dell’Accademia Gioenia de scienze naturali in Catania 12: 369-376.

[B44] Treadwell AL (1901) The Polychaetous annelids of Porto Rico. Bulletin of the United States Fish Commission 20 (2): 181-210.

[B45] Uchida H (2004) Hesionidae (Annelida, Polichaeta [sic]) from Japan. I. Kuroshio Biosphere 1: 27–92, Plate 1.

[B46] Uschakov PV (1955) Polychaeta of the far eastern seas of the U.S.S.R: Moscow & Leningrad, Izdatel’stvo Akademii Nauk SSSR. [in Russian]

[B47] Verrill AE (1873) Report upon the invertebrate animals of Vineyard Sound and the adjacent waters, with an account of the physical characters of the region. United States Commission of Fish and Fisheries Annual Reports 1871–1872: 295–778.

[B48] Viéitez JM Alós C Parapar J Besteiro C Moreira J Núñez J Laborda AJ San Martín G (2004) Annelida Polychaeta I. In: Ramos MA et al. (Eds) Fauna Ibérica, Vol. 25. Museo Nacional de Ciencias Naturales, CSIC, Madrid, 530 pp.

[B49] von Marenzeller E (1874) Zur Kenntniss der adriatischen Anneliden. Sitzberichte der Akndemie der Wissenschaften in Wien 69: 407-482.

[B50] Wagler J (1830) Natürliches System der Amphibien, mit vorangehender Classification der Säugthiere und Vögel. Ein Beitrag zur Vergleichenden Zoologie. Münich, Stuttgart and Tübingen: J.G. Cottas’schen Buchhandlung 354 pp. doi: 10.5962/bhl.title.58730

[B51] Willey A (1905) Report on the Polychaeta collected by Professor Herdman at Ceylon, in 1902. Report to the Government of Ceylon on the Pearl Oyster Fisheries of the Gulf of Manaar 4: 243-324.

